# The *RELN* heterozygous single-nucleotide polymorphism rs362691 increases the prefrontal cortical thickness and modulates systemizing-related autistic tendencies in typically developing children and adolescents

**DOI:** 10.3389/fnins.2025.1574700

**Published:** 2025-06-06

**Authors:** Hiroki Sato, Mitsunari Abe, Hikaru Takeuchi, Hiroaki Tomita, Shigeo Kure, Ryuta Kawashima, Yasuyuki Taki

**Affiliations:** ^1^Department of Pediatrics, Tohoku University Graduate School of Medicine, Sendai, Miyagi, Japan; ^2^Children's Center, Natori, Miyagi, Japan; ^3^Department of Aging Research and Geriatric Medicine, Institute of Development, Aging and Cancer, Tohoku University, Sendai, Miyagi, Japan; ^4^Department of Advanced Neuroimaging, Integrative Brain Imaging Center, National Center of Neurology and Psychiatry, Tokyo, Japan; ^5^Division of Developmental Cognitive Neuroscience, Institute of Development, Aging and Cancer, Tohoku University, Sendai, Miyagi, Japan; ^6^Department of Psychiatry, Graduate School of Medicine, Tohoku University, Sendai, Miyagi, Japan; ^7^Miyagi Children’s Hospital, Sendai, Miyagi, Japan

**Keywords:** reelin, polymorphism, empathizing-systemizing theory, behavioral trait, magnetic resonance imaging, morphology

## Abstract

Reelin, a glycoprotein, plays an essential role in the development and maturation of neural circuits in the cerebral cortex during embryonic and postnatal stages. Animal and human studies suggest that insufficient reelin signaling due to RELN mutations may alter the functional properties of the prefrontal cortex and contribute to cortical dysplasia in the frontal and temporal lobes. A heterozygous missense mutation in RELN, rs362691 (p. Leu997Val), has been proposed to increase susceptibility to autism spectrum disorder (ASD). Based on the empathizing–systemizing theory, this study examined whether the rs362691 variant affects cortical thickness and modulates autism-related cognitive traits in typically developing children and adolescents. We hypothesized that individuals carrying the heterozygous Val/Leu genotype would exhibit greater prefrontal cortical thickness than those with the Val/Val genotype, and that this morphological difference would correlate with autistic cognitive traits. We also explored potential thickness differences in the frontal and temporal cortices. Our results showed that the heterozygous Val/Leu group did not differ from the Val/Val group in empathizing or systemizing trait scores. However, individuals with the Val/Leu genotype exhibited increased cortical thickness in the medial prefrontal sulci, which correlated with individual differences in systemizing traits. No significant association was observed between cortical thickness and empathizing traits across the whole brain. Additionally, greater cortical thickness was observed in the right superior temporal sulcus (STS), although this morphological difference was not associated with empathizing or systemizing traits. These findings suggest that while the rs362691 variant does not significantly influence autism-related cognitive styles per se, it may alter cortical morphology in prefrontal regions functionally linked to systemizing traits in typically developing individuals. Several methodological limitations in the employed data should be considered. Future studies with larger, age-appropriate cohorts and standardized personality measures will be necessary to validate and extend these findings.

## 1 Introduction

Reelin glycoprotein, encoded by the reelin gene (*RELN*), plays an essential role in the formation of vertical laminar structures of the cerebral cortex during the embryonic period and also participates in the maturation of intracortical circuits in the postnatal stage (Jossin, 2020; Rice and Curran, 2001; Tissir and Goffinet, 2003; Wasser and Herz, 2017). Patients with autosomal recessive lissencephaly with cerebellar hypoplasia and the mutant reeler mouse harbor homozygous nonsense mutations in the *RELN* gene, resulting in a complete loss of reelin signaling and the development of lissencephaly (Hong et al., 2000). Heterozygous nonsense mutations of the *RELN* impair the morphofunctional development of neural circuits in the prefrontal cortex (Iafrati et al., 2014). Juvenile mice carrying these mutations exhibited behavioral abnormalities resembling psychiatric symptoms (Iafrati et al., 2014). These findings suggest that heterozygous *RELN* variants may be associated with pathological development of the prefrontal cortex (Iafrati et al., 2014) due to insufficient secretion of reelin protein (Lammert et al., 2017). Genetic evidence suggests that mutations in the *RELN* locus are associated with various neuropsychiatric disorders, including autism spectrum disorder or schizophrenia (Ishii et al., 2016). It has been proposed that missense mutations in the *RELN*, particular single nucleotide polymorphisms (SNPs), might contribute to the manifestation of abnormal behaviors characteristic of autism spectrum disorder (ASD) (Serajee et al., 2006; Wang et al., 2014). Meta-analyses suggest that the *RELN* SNP rs362691 (p. Leu997Val) may confer a greater susceptibility to ASD than other *RELN* variants (Wang et al., 2014). The *RELN* rs362691 is a missense variant within the reelin repeat domains. Missense mutations within the reelin repeat domains result in insufficient reelin signaling (Lammert et al., 2017). However, it is unknown whether the *RELN* SNP rs362691 would alter morphological development or maturation of cortical circuits and thus would exhibit autistic traits. We hypothesized that insufficient secretion of reelin protein in individuals with the heterozygous rs362691 genotype (Val/Leu) may lead to differences in prefrontal cortical thickness compared to individuals with the Val/Val genotype. Furthermore, the differences in cortical thickness would be associated with autistic traits. In the present study, we examined the relationship between cortical morphology and autistic traits in typically developing children and adolescents with the *RELN* SNP rs362691. Human and animal models with the homozygous nonsense mutations of the *RELN* reveal cortical malformations that are most pronounced in the frontal and temporal lobes (Hong et al., 2000). It is unknown whether the *RELN* SNP rs362691 would exhibit cortical malformations in the frontal or temporal cortex. We predicted higher cortical thickness in the frontal or temporal cortex in typically developing children and adolescents with the *RELN* SNP rs362691. We compared the cortical thickness across the whole brain between the heterozygous Val/Leu and Val/Val allele groups. To this end, we chose data from a children’s imaging cohort study comprising anatomical brain magnetic resonance imaging (MRI) data, DNA samples, and a questionnaire-based assessment of autistic traits (Sassa et al., 2012). We used the children’s versions of the Systemizing and Empathy Quotients (SQ-C and EQ-C, respectively) to evaluate autistic traits with respect to two fundamental cognitive domains: empathizing and systemizing (Auyeung et al., 2009; Baron-Cohen et al., 2003; Baron-Cohen and Wheelwright, 2004). Empathizing is the mental drive to understand affective states in other individuals and react to them with a mindful attitude. Systemizing is the mental drive for analyzing and constructing rule-based systems (Baron-Cohen et al., 2003; Baron-Cohen and Wheelwright, 2004). Patients with ASD tend to show lower scores on the Empathy Quotient (EQ), reflecting reduced empathizing traits, and higher scores on the Systemizing Quotient (SQ), indicating stronger systemizing tendencies. Empathizing engages neural networks related to empathy, mentalizing, and theory of mind (Baron-Cohen and Wheelwright, 2004), including the medial prefrontal cortex, anterior cingulate cortex, insula, inferior frontal gyrus, temporoparietal junction, and amygdala. Systemizing involves networks associated with executive functions, reasoning, and abstract thinking, including the posterior parietal cortex, medial and lateral prefrontal cortex, angular gyrus, and basal ganglia. Our results revealed that, in typically developing individuals, the *RELN* SNP rs362691 did not reduce empathizing traits or increase systemizing traits. We found increased cortical thickness in medial prefrontal regions associated with interindividual differences in systemizing tendencies related to autistic traits in individuals carrying this SNP. Additionally, increased cortical thickness was observed in the right superior temporal sulcus (STS), which was also unrelated to autistic traits. We conclude that the *RELN* single-nucleotide polymorphism rs362691 modulates autistic traits related to systemizing, possibly by increasing cortical thickness in the prefrontal sulci of typically developing children and adolescents.

## 2 Materials and methods

### 2.1 Participants

**Figure 1:**
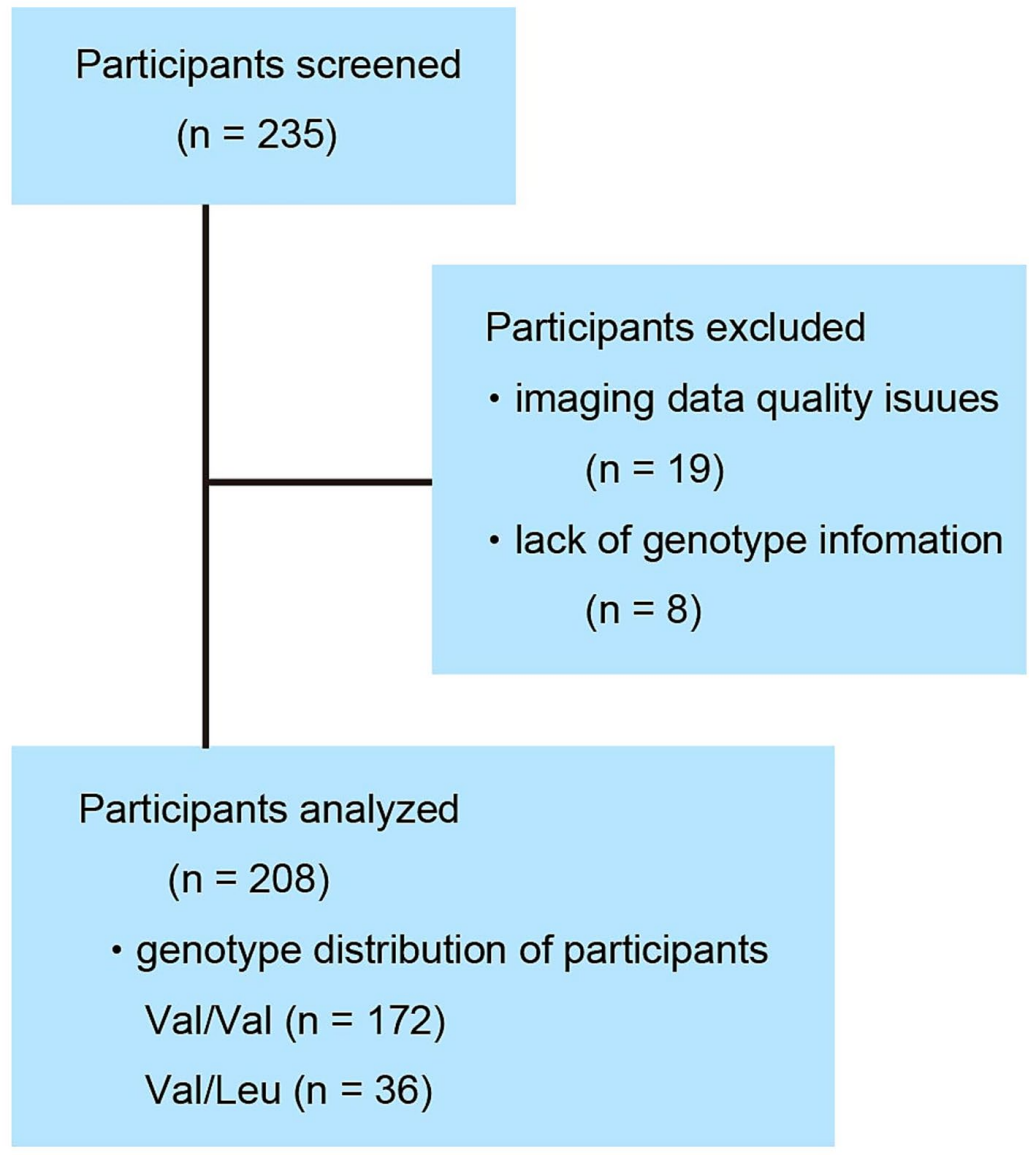
Participant disposition. Of the 235 individuals surveyed, 27 were excluded due to insufficient data quality. Among the final 208 participants included in the analysis, 172 were homozygous for the Val/Val genotype and 36 were heterozygous for the Val/Leu genotype.

Data regarding brain MRI scans and neuropsychological tests were obtained from 235 typically developing individuals. Due to cortical parcellation errors in 19 individuals and missing *RELN* genotype information in 8 individuals, a total of 27 individuals were excluded from the analysis. Imaging analyses were performed in 208 individuals (55 were children, 129 were adolescents, and 24 were young adults, 104 males and 104 females, age range, 8.35–20.61 years with a mean ± standard deviation of 14.27 ± 2.99 years). Finally, we acquired data of 36 in the Val/Leu group and 172 individuals in the Val/Val group (Figure 1). All participants were recruited through voluntary enrollment, and informed consent was obtained prior to participation in the study. Data were collected in the following order: participants completed a set of questionnaires at home, then underwent neuropsychological and genetic testing, followed by an MRI examination during a single-day visit. Data were collected between April 2008 and March 2014. All enrolled individuals were healthy, right-handed, of Japanese nationality, and had no history of malignant tumors or head trauma involving the loss of consciousness. The Edinburgh Handedness Inventory was used to assess right-handedness (Oldfield, 1971). This study was conducted in accordance with the Declaration of Helsinki (World Medical Association, 1991). After a complete explanation of the purpose and procedures of the study, written informed consent was obtained from each individual and their parent prior to MRI scanning, gene testing, neuropsychological testing, and questionnaire (SQ-C, EQ-C, and the Edinburgh Handedness Inventory) completion. A special version of the informed consent form was used to obtain informed consent from the children. This study was approved by the Institutional Review Board of Tohoku University.

### 2.2 Neuropsychological testing

Intelligence was assessed using the Japanese version of the Wechsler Adult Intelligence Scale-Third Edition (WAIS-III) or the Wechsler Intelligence Scale for Children-Third Edition (WISC-III). The WAIS-III was used for individuals aged ≧16 years, and the WISC-III was used for those aged <16 years. None of the enrolled individuals had a full-scale intelligence quotient (FSIQ) of <70.

**Figure 2:**
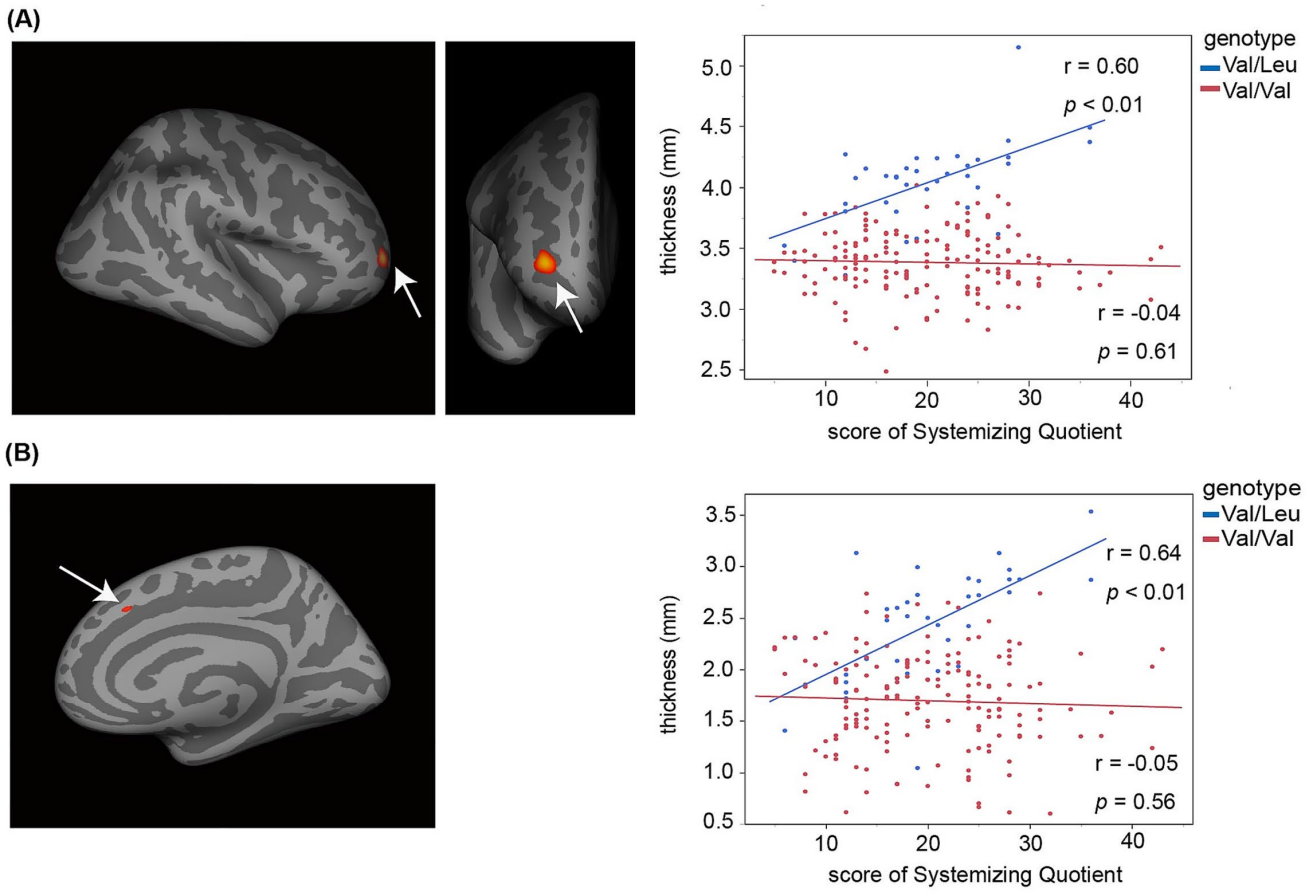
Increased cortical thickness in prefrontal cortical regions was associated with individual differences in autism-related systemizing cognitive traits in the Val/Leu genotype group, but not in the Val/Val group. The scatter plots display the correlation between SQ-C scores (*x*-axis) and cortical thickness (*y*-axis) in two prefrontal subregions. Blue dots represent individual data points from the Val/Leu group, while red dots represent those from the Val/Val group. **(A)** Right rostral part of the middle frontal sulcus. **(B)** Right middle part of the superior frontal sulcus.

**Figure 3:**
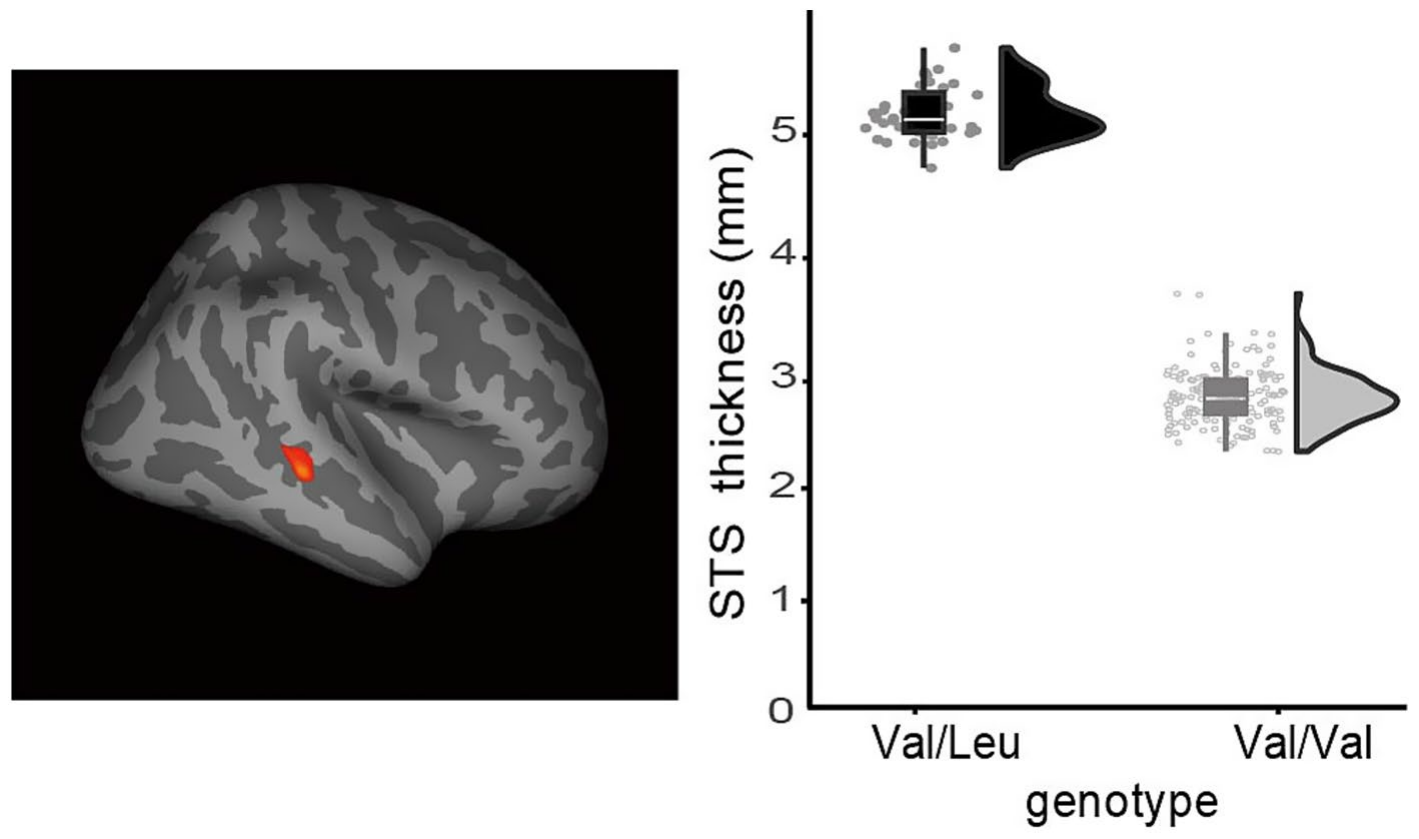
Typically developing individuals with the RELN rs362691 Val/Leu genotype exhibited greater cortical thickness in the right superior temporal sulcus (STS) compared to those with the Val/Val genotype. A lateral view of the right hemisphere is shown. Increased STS cortical thickness was observed in the Val/Leu group relative to the Val/Val group. The statistical threshold was set at FDR-corrected *p* < 0.05.

We also analyzed parent-completed EQ-C and SQ-C scores as measures of cognitive traits (Auyeung et al., 2009). Parents reported lower scores on the EQ-C and higher scores on the SQ-C when assessing children with autism spectrum disorder compared to typically developing children. No clear cutoff values have been established. Originally, the children’s version is administered to participants of children’s age. However, in our employed cohort study, the EQ-C and SQ-C were administered to all individuals across a broad age range, including children, adolescents, and young adults. The adult versions of the EQ and SQ were not administered to adolescents or young adults. A previous study reported no overall discrepancies between self- and parent-report of the EQ or SQ in typically developing children and adolescents (Johnson et al., 2009). We therefore assumed that the participants’ autistic traits can be estimated from their parents’ reports across all participants’ ages. We included participants of all ages—including children, adolescents, and young adults—in the main analyses using the EQ-C and SQ-C (Figures 2, 3). See the information in the Method subsection, 2.6 MRI data preprocessing and statistical analyses.

The original aim of our cohort study was to investigate the development of cortical morphology and the genetic background underlying personality traits, as characterized by the Big Five and EQ–SQ models, in typically developing individuals. Questionnaires designed to assess autistic behaviors—such as the Autism Spectrum Quotient (AQ), Autism Behavior Checklist (ABC), or Social Responsiveness Scale (SRS)—were not administered in this cohort.

### 2.3 Genotyping

High-molecular-weight DNA was isolated from the saliva of the enrolled individuals using Oragene containers (DNA Genotek Inc., Ottawa, ON, Canada) according to the manufacturer’s protocol. Samples were genotyped for the *RELN* SNP rs362691 (p. Leu997Val) using TaqMan analysis (assay ID: C_339123_1; Applied Biosystems, Foster City, CA, USA). Genotyping was performed using a 10-μL sample volume containing 20 ng of genomic DNA, 5 μL of TaqMan Mastermix (Applied Biosystems), 0.125 μL of TaqMan assay reagent, and 2.375 μL of H_2_O. Genotyping was performed on a CFX96™ Real-Time Polymerase Chain Reaction Detection System, and the resultant genotypes were scored using the algorithm and software supplied by the manufacturer (BioRad, Hercules, CA, USA). Genotyping assays were validated by duplicate measurements, and blanks were used as quality controls for all measurements. The nucleotide variants of rs362691 are G/G, G/C, and C/C. The G allele corresponds to the amino acid Valine (Val), and the C allele to the amino acid Leucine (Leu). The substitution from G to C occurs at a frequency of 2.27–9.74% in East Asian or Japanese populations (National Center for Biotechnology Information dbSNP database, https://www.ncbi.nlm.nih.gov/snp/rs362691: the Japanese Multi-Omics Reference Panel, https://jmorp.megabank.tohoku.ac.jp). Theoretically, we expected very few individuals with the homozygous C/C (Leu/Leu) genotype. Indeed, no participants with this genotype were observed in the present study.

### 2.4 Image acquisition

All images were acquired using a 3-T Philips Intera Achieva scanner (Philips, Amsterdam, The Netherlands). Three-dimensional, high-resolution, T1-weighted images were obtained using a magnetization-prepared rapid gradient-echo sequence using the following parameters: matrix = 240 × 240, repetition time = 6.5 ms, echo time = 3 ms, inversion time = 711 ms, field of view = 24 cm, slices = 162, slice thickness = 1.0 mm, and scan duration = 8 min and 3 s.

### 2.5 Statistical analysis of the demographic data

Demographic information (Table 1) was compared between the heterozygous Val/Leu and the Val/Val allele groups. We conducted chi-square tests for the categorical variable (Male/Female) or unpaired t-tests for continuous variables (Age, FSIQ, SQ-C or EQ-C). Statistics were performed using the JMP Pro 17.1.0 software (JMP Statistical Discovery LLC, Cary, NC, USA).

**Table 1:** Demographic characteristics of the study participants.

Variable	Val/Leu	Val/Val	*p*
*N*	36	172	
Male/Female	21/15	83/89	>0.05
Age (mean ± SD)	14.39 ± 2.64	14.25 ± 3.07	0.79
FSIQ (mean ± SD)	102.42 ± 11.95	104.9 ± 12.14	0.27
SQ-C (mean ± SD)	20.08 ± 7.02	19.79 ± 7.82	0.84
EQ-C (mean ± SD)	32.58 ± 7.55	31.25 ± 8.54	0.39

FSIQ, full-scale intelligence quotient; SQ-C, children’s versions of the Systemizing Quotients; EQ-C, children’s versions of the Empathy Quotients.

### 2.6 MRI data preprocessing and statistical analyses

We used the free-distributed software FreeSurfer (version 5.3.0) to compute cortical thickness from anatomical MRI data. The FreeSurfer software enables cortical thickness estimation, cortical parcellation, and statistical analysis of the entire cortex from the brain imaging data (Fischl et al., 1999, 2000, 2002). The processing pipeline includes correction for magnetic field inhomogeneities and skull stripping to remove non-brain tissue. The white matter is segmented, a mesh representation of the white matter surface is generated, and the white matter surface is subsequently smoothed (Clarkson et al., 2011). Topological defects are corrected automatically. Following the initial surface model construction, a secondary smoothing procedure is applied to achieve a realistic representation of the gray matter/white matter boundary. This gray matter/white matter boundary is expanded to the pial surface utilizing a deformable surface algorithm. Hence, each mesh node on the white matter surface has a corresponding node on the pial surface. To measure cortical thickness and compare cortical thickness between subjects, data are registered to an average spherical surface, and the shortest distance between points on the gray matter/white matter boundary and the pial surface, and vice versa, is calculated and averaged (Fischl et al., 1999, 2000). The registration and segmentation of MR images are independent of the imaging process physics (Fischl et al., 2004; Destrieux et al., 2010), and the intensity model of an existing atlas is automatically adjusted to the new image data (Han and Fischl, 2007). Each individual’s cortex was visually inspected and manually corrected when necessary. To minimize signal noise, each subject’s surface maps were smoothed using a 10 mm full-width at half the maximum Gaussian kernel.

A cross-subject general linear model (GLM) was employed to estimate cortical thickness associated with the two rs362691 genotypes: the heterozygous Val/Leu and the homozygous Val/Val alleles. To test our hypothesis, we examined whether the heterozygous rs362691 genotype modulates autistic-like traits in association with altered cortical thickness in the prefrontal cortex. We conducted vertex-wise correlation analyses between cortical thickness and cognitive trait scores (EQ-C and SQ-C) within each genotype group, controlling for age, sex, and total intracranial volume (Figure 2). In the SQ-C analysis, EQ-C scores were additionally included as a covariate of no primary interest. These analyses were performed across the entire cortical surface. Significant foci were observed in the prefrontal cortex where cortical thickness correlated with inter-individual variability in systemizing traits. These foci were defined as regions of interest (ROIs), and further ROI-based analyses were conducted. We performed partial correlation analyses to validate the relationship between cortical thickness and behavioral traits, while controlling for interregional correlations between the two prefrontal ROIs. We further compared cortical thickness in these ROIs between the Val/Leu and Val/Val groups using unpaired t-tests. In addition, we explored the presence of cortical dysplasia in the frontal and temporal cortices of typically developing individuals with the Val/Leu genotype, given that dysplasia has been reported in cases with homozygous nonsense mutations in *RELN*. To assess potential group differences, we conducted vertex-wise unpaired t-tests comparing cortical thickness between the two genotype groups (Figure 3). For this analysis, estimated cortical thickness values were adjusted for age, sex, and total intracranial volume.

Evidence suggests that cortical thickness increases and then decreases during postnatal development, particularly between infancy and adolescence. We further examined whether the effects of genotype on cortical thickness, as observed in Figures 2, 3, differed between younger and older participants with the heterozygous rs362691 Val/Leu genotype. Both the Val/Leu and Val/Val genotype groups were divided into two age-based subgroups: younger (<14 years) and older (≥14 years). For Figure 2, we constructed regression models using participants’ paired data of SQ-C scores and cortical thickness in the prefrontal ROIs, and compared the regression slopes between the Val/Leu and Val/Val groups. A homogeneity-of-slopes test, typically performed prior to analysis of covariance (ANCOVA), was conducted to test for interaction effects between genotype and SQ-C on cortical thickness. In addition, we examined whether the higher cortical thickness in the right superior temporal sulcus (STS), as shown in Figure 3, differed by age in individuals with the Val/Leu genotype. A 2 × 2 analysis of variance (ANOVA) was performed with genotype (Val/Leu vs. Val/Val) and age group (<14 vs. ≥14) as factors. While the main effect of genotype was already confirmed in the whole-brain analysis (Figure 3), here we tested for both the interaction effect between genotype and age, and the main effect of age. For whole-brain analyses, statistical significance was corrected for multiple comparisons using the false discovery rate (FDR) method at *p* < 0.05 (Genovese et al., 2002). For ROI-based analyses, a Bonferroni-corrected threshold of *p* < 0.05 was applied where necessary.

## 3 Results

### 3.1 Genotyping and neuropsychological testing

The rs362691 genotype was determined for all participants. Among the enrolled individuals, 36 were heterozygous for the Val/Leu allele (Figure 1), and none were homozygous for the Leu/Leu allele. A total of 172 individuals were homozygous for the Val/Val allele. The genotype distribution did not deviate significantly from Hardy–Weinberg equilibrium (*χ*^2^ = 1.87, *p* = 0.91). The Val/Leu and Val/Val allele groups showed comparable performance across several cognitive batteries administered in this study (Table 1). No significant group differences were observed in either SQ-C or EQ-C scores. Furthermore, the rs362691 SNP was not associated with differences in cognitive domains related to general intelligence. These findings suggest that the *RELN* heterozygous rs362691 polymorphism is unlikely to account for variation at the extreme end of the empathizing–systemizing spectrum commonly observed in individuals with ASD.

### 3.2 Cortical thickness associated with interindividual variability in the empathizing-systemizing cognitive traits

We conducted vertex-wise correlation analyses between cortical thickness and cognitive trait scores (EQ-C and SQ-C) across the whole brain. In the heterozygous Val/Leu group, greater SQ-C scores were significantly associated with increased cortical thickness in two prefrontal regions: the rostral part of the middle frontal sulcus (coordinates: *x* = 27.6, *y* = 48.3, *z* = 2.7; FDR-corrected *p* < 0.05) and the middle part of the superior frontal sulcus (coordinates: *x* = 9.0, *y* = 22.9, *z* = 39.4; FDR-corrected *p* < 0.05). No such associations were observed in the homozygous Val/Val group (Figure 2). These two regions fall within the anatomical definition of the medial prefrontal cortex (Samara et al., 2017). Animal models carrying *RELN* heterozygous mutations suggest impairments in the morphofunctional development and maturation of prefrontal cortical circuits, accompanied by psychiatric-like behaviors. It is therefore plausible that a missense variant of *RELN* may alter cortical morphology in the medial prefrontal cortex and underlie interindividual differences in autism-related systemizing traits within the typical population range (Baron-Cohen and Lombardo, 2017). Cortical thickness values in the two prefrontal regions were moderately correlated (*r* = 0.44, *p* = 0.01). To validate the vertex-wise findings shown in Figure 2, we conducted ROI-based partial correlation analyses, controlling for interregional correlations between the two prefrontal regions. The associations between cortical thickness and SQ-C scores remained significant even after controlling for interregional effects (rostral part of the middle frontal sulcus: *r* = 0.47, *p* = 4.40 × 10^−3^; middle part of the superior frontal sulcus: *r* = 0.52, *p* = 1.30 × 10^−3^). These results support the notion that cortical morphology in these prefrontal foci underlies individual differences in systemizing traits. We also performed ROI-based comparisons to test whether cortical thickness differed between the Val/Leu and Val/Val groups in these regions. The Val/Leu group showed greater cortical thickness than the Val/Val group in both regions examined (rostral part of the middle frontal sulcus: *t* = 13.10, *p* = 6.64 × 10^−29^; middle part of the superior frontal sulcus: *t* = 8.52, *p* = 3.32 × 10^−15^).

In contrast, neither the Val/Leu nor the Val/Val group showed any significant associations between cortical thickness and EQ-C scores across the whole brain (FDR-corrected *p* > 0.05).

### 3.3 Intergroup differences in cortical thickness by genotype

We examined whether the rs362691 polymorphism, specifically the heterozygous Val/Leu genotype of *RELN*, is associated with abnormal cortical morphology in the frontal or temporal cortex that is unrelated to autism-related cognitive traits. We conducted vertex-wise analyses to compare cortical thickness between the heterozygous Val/Leu and homozygous Val/Val genotype groups. The plot presents estimated cortical thickness values after adjusting for covariates (see Methods). The Val/Leu group exhibited significantly greater thickness in the right superior temporal sulcus (STS) at MNI coordinates *x* = 45.2, *y* = −31.3, *z* = −6.6, compared to the Val/Val group (FDR-corrected *p* < 0.05; Figure 2). No other regions showed significant group differences, even at a liberal threshold of uncorrected *p* < 0.05, except for the two prefrontal foci already identified in Figure 2. The identified STS region corresponds to the posterior STS (Ochiai et al., 2004). This area is not adjacent to the temporoparietal junction and is therefore unlikely to overlap with key regions commonly implicated in clinical phenotypes of ASD. Prior studies have reported that the posterior STS is a hub for integrating visual and auditory information (Deen et al., 2015).

### 3.4 The genotype effects of the heterozygous Val/Leu allele on cortical thickness by age

Evidence suggests that cortical thickness increases and then decreases during postnatal development, particularly between infancy and adolescence (Bethlehem et al., 2022). We further examined whether the effects of genotype on cortical thickness, as already observed in Figures 2, 3, differed between younger and older participants with the heterozygous rs362691 Val/Leu genotype. Both the Val/Leu and Val/Val genotype groups were divided into two age-based subgroups: a younger subgroup (<14 years) and an older subgroup (≥14 years), each consisting of 18 individuals. For Figure 2, we constructed regression models using participants’ paired data of SQ-C scores and cortical thickness in the prefrontal ROIs, and compared the regression slopes between the Val/Leu and Val/Val groups. A homogeneity-of-slopes test (see Methods) was conducted to assess interaction effects between genotype and SQ-C scores on cortical thickness. None of the homogeneity tests reached statistical significance in either of the two ROIs, regardless of genotype (*p* > 0.4 for all tests; see Supplementary material 2). These results suggest that the effect of the heterozygous rs362691 Val/Leu genotype on the cortical thickness and the systemizing traits does not differ across adolescent age groups. In addition, we examined whether the higher cortical thickness in the right superior temporal sulcus (STS), as shown in Figure 3, differed by age in individuals with the Val/Leu genotype. A 2 × 2 analysis of variance (ANOVA) was performed with genotype (Val/Leu vs. Val/Val) and age group (<14 vs. ≥14) as factors. While the main effect of genotype was already confirmed in the whole-brain analysis (Figure 3), here we tested for both the interaction effect between genotype and age, and the main effect of age. No significant main effect of age or genotype-by-age interaction was observed (*p* > 0.8 for all tests; see Supplementary material 1). These results suggest that the genotype effect on right STS cortical thickness does not vary significantly across adolescent age groups. We found no evidence that the effect of the heterozygous rs362691 Val/Leu genotype on cortical thickness differed across adolescent participants. Notably, adolescents comprised the largest proportion of our sample (129 out of 208 participants). However, we did not have sufficient data to evaluate genotype-by-age interaction effects on cortical morphology in early childhood.

## 4 Discussion

Our results indicate that the heterozygous Val/Leu allele of *RELN* rs362691 does not influence general intelligence or empathizing–systemizing cognitive traits in typically developing individuals. However, this allele is associated with increased cortical thickness in medial prefrontal cortex foci, which in turn, correlate with individual differences in autism-related systemizing traits in children and adolescents. These findings suggest that rs362691 may play a role in modulating prefrontal cortical morphology and shaping personality traits, particularly systemizing tendencies, within the typical population. Hyperplasia in the middle portion of the right superior temporal sulcus (STS) was also observed in participants carrying the heterozygous allele. However, this morphological anomaly was not associated with empathizing–systemizing cognitive tendencies.

Several meta-analyses have supported the notion that the heterozygous Val/Leu genotype of *RELN* rs362691 is associated with increased susceptibility to autism spectrum disorders (ASD). However, no prior studies have reported a relationship between this genotype and cortical morphology linked to autism-related traits. Our findings revealed that increased cortical thickness in medial prefrontal foci was associated with individual differences in systemizing traits among typically developing children and adolescents carrying the Val/Leu genotype of rs362691. The identified prefrontal regions correspond anatomically to the medial parts of Brodmann areas 7 and 10. These regions have been implicated in cognitive functions such as reasoning, abstract thinking, and logical inference (Baron-Cohen and Lombardo, 2017; Demetriou et al., 2018; Houdé et al., 2010; Rivera et al., 2005), which conceptually align with the systemizing trait. Previous evidence has also shown that increased cortical thickness in the dorsomedial prefrontal cortex may underlie higher systemizing tendencies in typically developing males compared to females (Lai et al., 2012). Our results reveal a behavioral–neural association between individual differences in systemizing tendencies and cortical morphology in the prefrontal cortex. Juvenile reelin-haploinsufficient heterozygous *reeler* mice, which carry heterozygous nonsense mutations in *RELN*, exhibit reduced dendritic spine density and abnormal long-term potentiation in the prefrontal cortex. These mice also display atypical perceptual responses and behaviors resembling those observed in schizophrenia and autism spectrum disorder (ASD) (Ishii et al., 2016). Both schizophrenia and ASD share actin-dependent pathological mechanisms that result in altered dendritic spine morphology and impaired synaptic plasticity (Borovac et al., 2018). Clinically, both conditions also feature overlapping behavioral phenotypes, including elevated systemizing tendencies. Heterozygous missense mutations within the reelin repeat domains are thought to result in insufficient reelin signaling. The Val/Leu genotype of *RELN* rs362691 represents a missense variant located within these repeat domains (Serajee et al., 2006). Our findings suggest that insufficient reelin signaling may contribute to individual variability in systemizing tendencies in typically developing individuals, possibly by modulating the morphofunctional properties of medial prefrontal circuits (Hong et al., 2000).

In our sample, neither the heterozygous Val/Leu nor the homozygous Val/Val genotype group showed a significant association between cortical thickness and empathizing traits across the whole brain. Previous studies have reported that typically developing children with higher empathizing tendencies exhibit greater cortical volume in regions associated with reward and empathy processing, such as the superior temporal gyrus, inferior frontal gyrus, and ventral striatum (Lai et al., 2012; Sassa et al., 2012). However, to our knowledge, no studies have identified an association between empathizing traits and cortical morphology in the medial prefrontal cortex, which is consistent with the present findings. We did not administer any standardized questionnaires for preclinical screening of social skills or repetitive behaviors typically associated with autism spectrum disorder (ASD), such as the Autism-Spectrum Quotient (AQ), Autism Behavior Checklist (ABC), or Social Responsiveness Scale (SRS). Therefore, in the present study, we were not able to examine whether the heterozygous Val/Leu genotype of *RELN* rs362691 affects cortical morphology related to autistic-like social or habitual behaviors.

Homozygous nonsense mutations in *RELN* have been reported in patients with autosomal recessive lissencephaly and cerebellar hypoplasia. These patients exhibit hyperplasia in the frontal and temporal cortices, characterized by disorganized neuronal migration and laminar disarrangement. However, to our knowledge, cortical dysplasia has not been documented in *RELN*-haploinsufficient heterozygous models, including rs362691. In patients with temporal lobe epilepsy, reduced reelin gene expression has been associated with disrupted compact cell layering, suggesting that decreased reelin signaling may contribute to morphological abnormalities in the temporal cortex. It is therefore possible that the heterozygous *RELN* rs362691 polymorphism may similarly influence cortical morphology, leading to the hyperplasia observed in the superior temporal sulcus (STS) in the present study. To date, no previous studies have reported cortical hyperplasia associated with heterozygous *RELN* polymorphisms. The present study observed cortical hyperplasia in the posterior superior temporal sulcus (STS) in the right hemisphere. This region receives high-level multisensory input from the anterior and middle portions of the lateral temporal cortex and frontal areas (Deen et al., 2015). A neuroimaging study by Deen et al. (2015) demonstrated a functional dissociation among distinct cortical areas along the STS, using a range of tasks involving social perception and cognition. These tasks included story comprehension, and perception of voices, faces, biological motion, and theory of mind. The hyperplastic region identified in our study likely corresponds to the area involved in facial or voice perception. Facial and voice perception are foundational components of social cognition (Beauchamp, 2015; Saitovitch et al., 2012). However, the EQ-C and SQ-C questionnaires used in the present study did not directly assess these perceptual abilities. Therefore, it is plausible that the observed increase in cortical thickness in the STS did not influence empathizing–systemizing trait scores in participants with the heterozygous Val/Leu genotype. Given this limitation, we cannot conclude whether the observed cortical hyperplasia in the STS modulates other aspects of social perception. In the present study, we analyzed the existing data from the imaging cohort study conducted by our group. A recent large-scale, multi-database study emphasized that findings based on small sample sizes tend to be inconsistent. Over the past decade, MRI studies investigating brain regions associated with clinical phenotypes in autism and other psychiatric disorders have typically included over 100 participants. We examined only 32 individuals with the heterozygous Val/Leu allele of rs362691 in the present study. Thus, the relatively small sample size in our research may make readers less confident in our results compared to more recent imaging studies. According to the Japanese Multi-Omics Reference Panel (https://jmorp.megabank.tohoku.ac.jp), the frequency of individuals carrying this allele is 9.74% in the Japanese population. We will need a dataset of more than one thousand individuals to collect 100 individuals carrying this allele for a more robust imaging analysis (Assmann et al., 2021). We explicitly acknowledge the necessity of replication to reinforce the validity of our conclusions. It should be noted that, in this study, the SQ-C and EQ-C were administered to all participants, including adolescents and young adults beyond the originally targeted childhood age range. Replication using the adult versions of the EQ and SQ will be necessary to validate the present findings. The effects of genotype on cortical morphology, as observed in Figures 2, 3, did not differ significantly between younger and older subgroups, regardless of genotype. These findings suggest that the effect of genotype on cortical thickness does not substantially change during adolescence. However, we did not examine genotype-by-age interactions in younger children under 8 years old. Previous studies in juvenile mice with a *RELN*-haploinsufficient heterozygous genotype have demonstrated morphological abnormalities emerging shortly after weaning, corresponding to early childhood in humans. Furthermore, evidence suggests that cortical thickness changes dynamically throughout postnatal development, starting at infancy (Bethlehem et al., 2022). Thus, it remains possible that genotype-by-age interactions may be more pronounced during earlier developmental stages. Another limitation of this study is the inability to fully control for potential confounding factors such as environmental influences and socioeconomic status, which may also contribute to variations in cortical thickness. Finally, the relationship between *RELN* function and the mechanisms underlying systemizing traits remains unclear. Further research is warranted to elucidate this association.

In conclusion, the *RELN* SNP rs362691 with the heterozygous Val/Leu allele did not significantly influence autism-related cognitive styles, as characterized by lower empathizing and higher systemizing traits. However, this polymorphism was associated with increased cortical thickness in the medial prefrontal cortex, which in turn correlated with individual differences in systemizing traits. In addition, hyperplasia was observed in the right superior temporal sulcus (STS), although this morphological change was not associated with empathizing-systemizing tendencies. These findings suggest that the heterozygous rs362691 polymorphism may alter cortical morphology and be linked to autism-related systemizing traits in typically developing individuals. However, several methodological limitations should be considered. Future studies that address these limitations will be necessary to reinforce and validate our findings.

## Data Availability

The datasets presented in this article are not readily available because the data are not publicly available due to privacy or ethical restrictions. Requests to access the datasets should be directed to Yasuyuki Taki, yasuyuki.taki.c7@tohoku.ac.jp.
