# Supplementary figures and images for: The RELN heterozygous single-nucleotide polymorphism rs362691 increases the prefrontal cortical thickness and modulates systemizing-related autistic tendencies in typically developing children and adolescents
Source: Front Neurosci. 2025 Jun 6;19:1574700. doi: 10.3389/fnins.2025.1574700 (PMC12179114; doi:10.3389/fnins.2025.1574700)

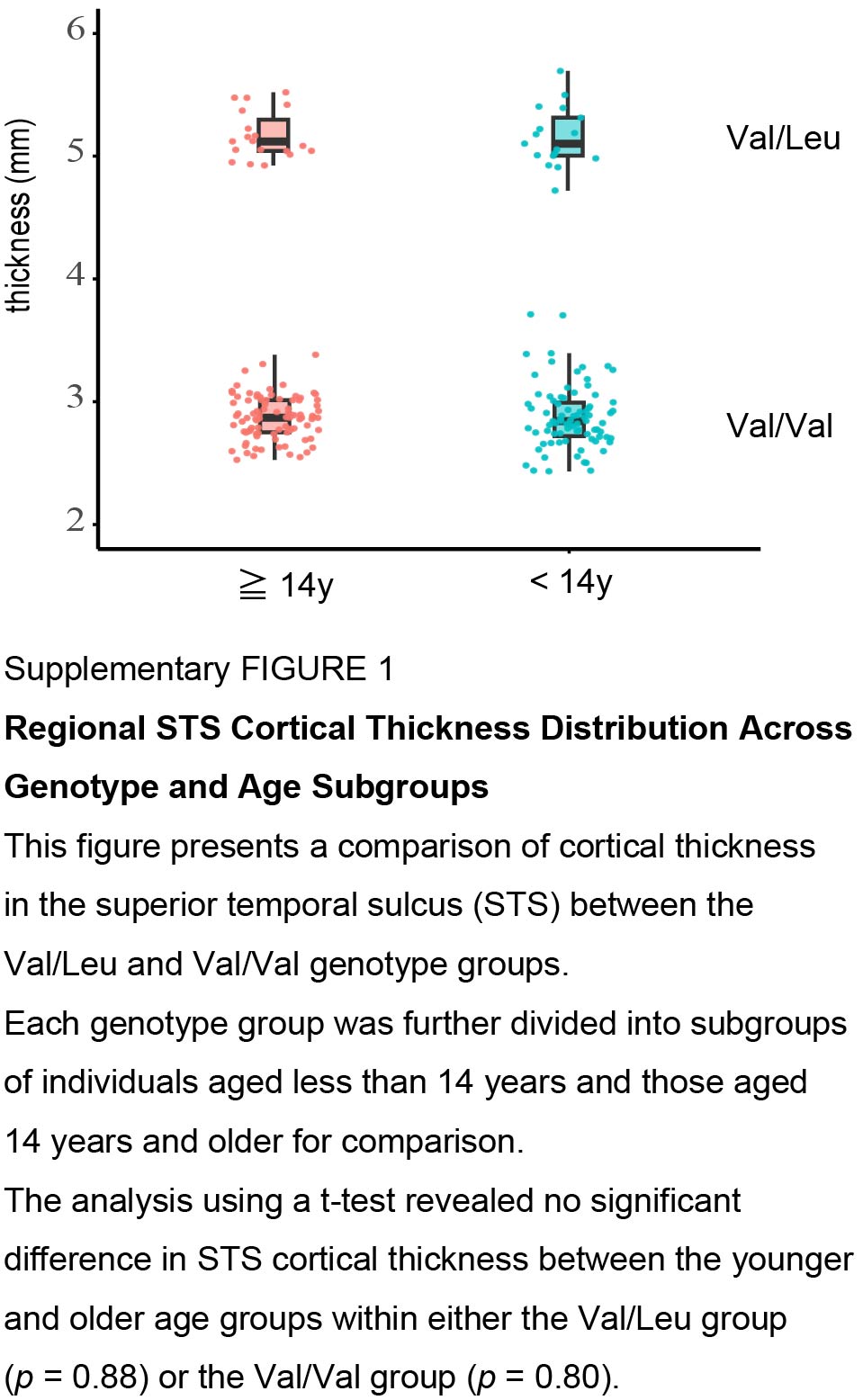

Supplement: Supplementary file 2 [file Image_1.jpeg]

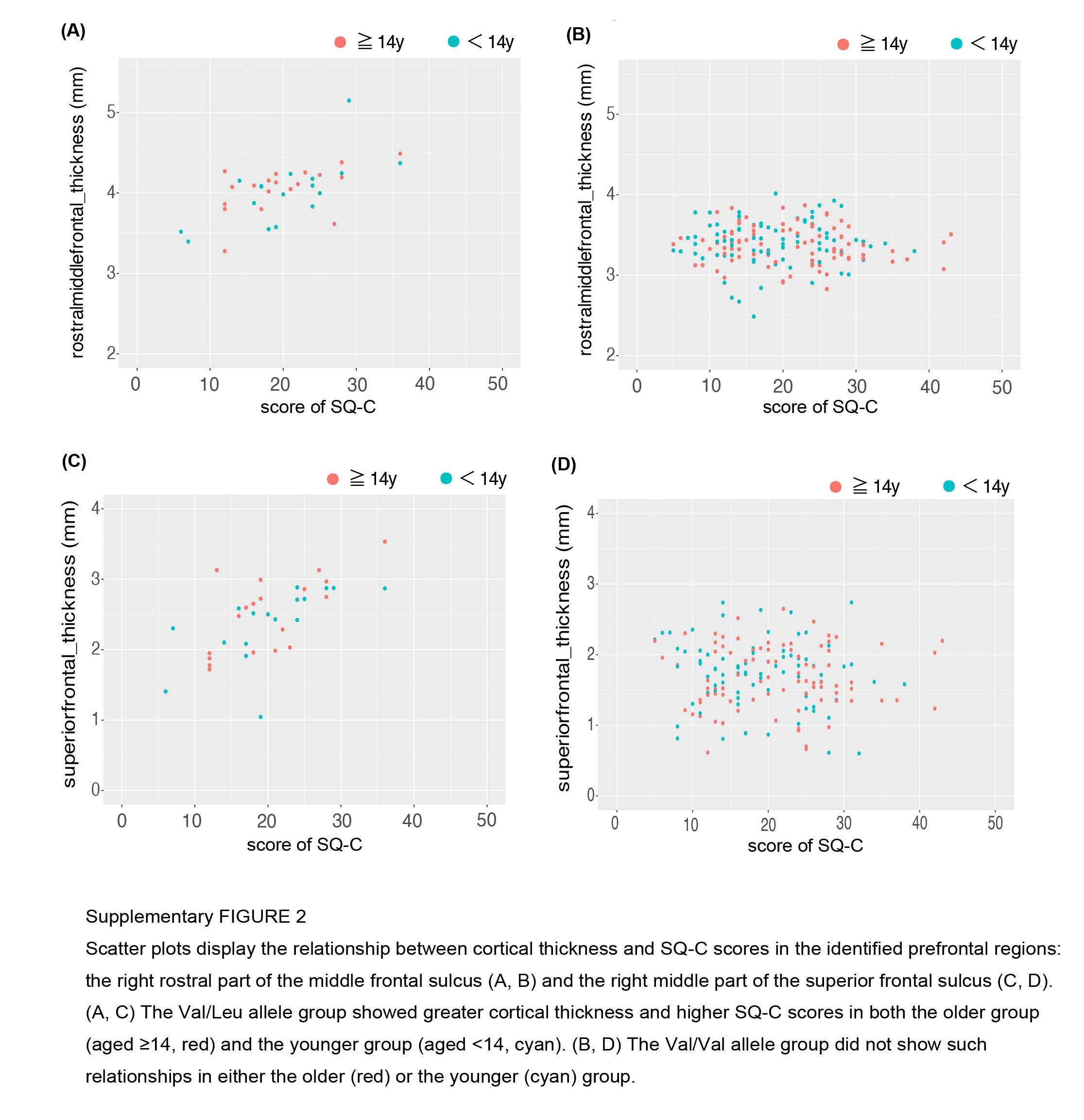

Supplement: Supplementary file 3 [file Image_2.jpeg]
